# Built-In Calibration Standard and Decision Support System for Controlling Structured Data Storage Systems Using Soft Computing Techniques

**DOI:** 10.1155/2022/3476004

**Published:** 2022-08-27

**Authors:** George Chellin Chandran, D. M. Mary Synthia Regis Prabha, P. Malathi, Dhiraj Kapila, M. S. Arunkumar, Devvret Verma, Dawit Mamiru Teressa

**Affiliations:** ^1^School of Computing Science and Engineering & Director Student Welfare, VIT-Bhopal University, Ashta, Madhya Pradesh 466114, India; ^2^Department of EEE, Noorul Islam Centre for Higher Education, Kanyakumari District, Kumaracoil, Tamil Nadu 629180, India; ^3^Department of Electronics and Communication Engineering, Prathyusha Engineering College, Tiruvallur, Chennai 602025, Tamilnadu, India; ^4^Department of Computer Science & Engineering, Lovely Professional University, Phagwara, India; ^5^School of Computing, Vel Tech Rangarajan Dr. Sagunthala R&D Institute of Science and Technology, Chennai 600062, Tamil Nadu, India; ^6^Department of Biotechnology, Graphic Era Deemed to be University, Dehradun 248002, Uttarakhand, India; ^7^Department of Chemical Engineering, College of Biological and Chemical Engineering, Addis Ababa Science and Technology University, Addis Ababa, Ethiopia

## Abstract

Several research that assesses, or assess computer systems has been undertaken in previous decades. Choosing an appropriate DBMS system in a computer application, though, was never completely arbitrary, based on the professional study. Developing a viable answer for such a challenge depending on business goals and needs from judgment necessitates a thorough study on information access as well as comprehensive professional evaluation. The research presents a DSS to help non-kinetics discover their proper DBMS solutions and otherwise information retention types faster. This suggested DSS is unique in that it uses MoSCoW to evaluate criterion weighting or deliberate, as well as assessment frameworks for quantifying overall levels of quasi criterion and ISO/IEC qualitative features to show the link between criterion based upon industry specialists' expertise. Companies that produce programs have difficulty integrating new innovations, such as Internet computing or information management platforms, into their operations. Because computer engineers and top programmers are usually specialists in their field, users need to consult or train other professionals. Therefore, computer development is an appropriate area for implementing judgment support technologies that can proactively help this prediction to choose the best technologies for their products. We offer a decision support system (DSS) to assist in the selection of the best appropriate information architecture. According to both example reports and specialists, this technique improves visibility into the choice method gives a deeper prioritized choice range than if customers have conducted their study individually, or saves the duration and expense of the judicial procedure.

## 1. Introduction

This procedure of evaluating the prospective worth of innovations and related contributions to the productivity and sustainability of systems supplying organizations (SPOs) is known as technological assessment [1]. Furthermore, one of the least important procedures for assessing creativity, attractiveness, and relevance of innovations to MSDSs is technological evaluation. As a result, selecting technologies is an important judgment step for SPOs. This task entails assessing and choosing the best appropriate technology for SPOs based on individual choices and needs [2, 3]. The choosing procedure is complicated since there are so many things to examine, including appropriateness and price. As a result, the technological choosing procedure may be represented as a cross-judgment multi-criteria decision-making (MCDM) issue which involves evaluating a collection of options while considering a collection of factors [4].

Authors propose to have developed and initiated a wide range of methods and toolkits to fix various technological challenging issues for SPOs in the latest days. There are several variants, but they all contain the critical stages of the judgment procedure [4]. The grading mechanism in its bulk of MCDM methods involves bilateral evaluation but is generally highly sustainable [5]. As a result, if the selection of options or requirements is changed, the entire assessment procedure should be redone. Such a process is expensive and only applies to a limited list of characteristics of criteria and options [6]. Choices about innovation use are frequently accepted on the fly, without regard to precision or audio techniques [7]. Additionally, because the findings of technological choice methods in the research are only relevant for a limited time, these must be repeated as knowledge progresses [8, 9]. As a result, to generate the best choice depending on its atmosphere's features, a recyclable fully evolved, and extensible judgment strategy is required [10].

This article presents a DSS to assist judgment in MCDM issues like database management systems choice [11]. The DSS is a device that may be employed throughout its living span or can find its recommendations in response to changing needs [12, 13]. The DSS uses seven different judgment procedure that creates a sustainable or upgradeable methodology for MCDM issues, and improves the reliability and trustworthiness of information collection [14]. All collections of criteria and options, as well as their relationships, for an MCDM challenge, may be updated and changed frequently while affecting the correctness of the choice system [15]. The DSS is distinctive in that it employs the MoSCoW resourcing method [16] to evaluate requirements barbells or decrease ambiguity, as well as evaluation designs, quantify the principles of quasi requirements and ISO/IEC top-notch facets to imply the connection between requirements based on website specialists' understanding [17–19].

A basic research technique used in Section 2 is described, as well as every exploration concept checking example investigations that were conducted.  Section 3 provides an overview of the research on computer platform choice as well as the multiple ways to tackle judgment challenges.  Section 4 discusses the planned DSS that connects the overall technological choice issue in SPOs. DSS is subsequently used to solve the database management system (DBMS) acquisition challenges in Section 5, with several examples used to assess and underline the importance of the methodology. Following that, Section 6 outlines the suggested technique and provides recommendations for further research.

## 2. Materials and Methods

One of the issues is trying to attack computer development companies that are often unfamiliar with an area in which companies have to make technological decisions regarding incorporation into the respective commodities. Its innovation choice task can be patterned as an MCDM issue which involves categorization, creating plans, and resolving an issue predicated on the collection of requirements: (1) determining the unbiased, (2) selecting characteristics, (3) selecting options, (4) selecting the technique of the weight, (5) trying to implement the integration technique, and (6) making decisions focused in outcomes.

To assist such businesses, we suggest a DSS built upon a six judgment procedure and developed utilizing computer theory. The DSS's objective is to discover appropriate options that meet a set of area characteristic constraints [20]. DSS was motivated by professional information acquired through the standard designing research process. In conversations lasting around 45 to 90 hours, 14 professionals (3 DSS specialists, 2 academicians, 5 computer engineers, as well as 4 system designers) evaluated the DSS. Subject specialists are rationally chosen based on actual knowledge and knowledge as stated in respective business profiles.

Next, three experimental hypothesis example scenarios are used to assess the DSS's effectiveness and utility. Every single technological choice within every computer package was a piece of evaluation. To assess its DSS, they conducted three similar example investigations at two SPOs. This scenario study spanned about a workday and comprised of (1) establishing the area features needs, (2) ranking these, and (3) evaluating the DSS viable options to our ideas.

Table 1 shows a selection of literature-based MCDM methods. To determine the weighting of criterion, most MCDM approaches employ bilateral comparisons. In the case of an issue with a large number of parameters *n*(*n* − 1)/2, *n*(*n* − 1)/2, analyses were required and determining evaluation takes longer and becomes increasingly difficult as the amount of criterion rises. AHP and FAHP are hardly sustainable techniques. Whenever the selection of options or criteria is changed, the entire assessment procedure must be repeated. Such approaches were expensive and only apply to a restricted amount of parameters and options. Within research, MCMD methods are mostly used to construct property grade characteristics for evaluating options. These investigations are usually suitable to particular example investigations. Moreover, the findings of such MCDM techniques are only applicable for a limited time, thus it would become obsolete as technology develops, as well as fresh upgrades.

All issues are subclasses of MCDM challenges, while the DBMS choice issue is one subset of a TCOs choice issue. A cross-choice supporting system (MCDSS) for computer element choosing is presented in [22]. This MCDSS assesses 51 commercially available elements versus 631 selection factors. These researchers defined measures enabling the statistical assessment on choice criterion or groups containing criteria, like important choice variables or effective requirements groups, and demonstrated its applicability to a collection of true choice situations [23]. Neither the planned DSS nor MCDSS includes a large variety of parameters to aid judgment in the overall technological choice process. We also employ the ISO/IEC 25010 (ISO, 2011) as a collection of performance characteristics as a guideline. The grading techniques used by us and this MCDSS vary significantly [24]. The MoSCoW is used by our DSS to determine the importance of a criterion. It also includes evaluation methods for determining the scores of quasi criterion like the price of options.

This work proposes a DSS that uses a six-step judgment procedure to create sustainable or upgradeable choice modeling for MCDM issues, therefore improving the reliability or trustworthiness of information collection [21]. Let Alt = [*x*_1_, *x*_2_,…, *x*_|alt|_] constitute a collection of marketplace options (technology). Furthermore, Fea = [*f*_1_, *f*_2_,…, *f*_|Fea|_].

Is a collection of topic characteristics that contains the least significant technological or quasi topic characteristics of the options, such that every *xϵ* alt. Any portion of this collection's characteristics is supported? Its objective is to discover a viable replacement that meets a collection of domain-specific criteria (collection needs), wherein requirement is a subset of features. In other terms, option is the best option for meeting area characteristic criteria and satisfying the decision preferences. Manufacturers in most cases, the single optimum answer for any MCDM issue does not exist, thus judgment preferences must be used to distinguish among options [25].

Database administration systems, the Prototype administration scheme, or its Dialogue Synthesis control framework were three core elements of a standard DSS [26]. The database administration system is a collection of domain-specific information about an MCDM challenge. This prototype administration platform is a compilation of MCDM-related principles, algorithms, and expertise. Another client frontend for interacting among judgment is its Dialogue Synthesis administration platform.

**Figure 1:**
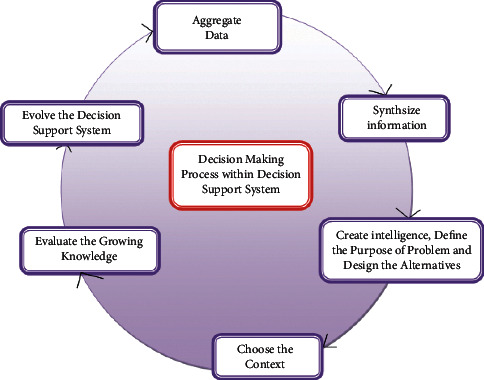
A model-based decision support system for technology selection issues.

The typical DSS interpretation engine insinuates answers rather than relying on information foundation truths and regulations, thus it may function autonomously of the remaining parts. For inputs, the reasoning machine takes area product needs or associated MoSCoW based priority via a dialogue synthesis control platform. This algorithm then searches a library of all modeling within the prototype administration computer for more regulations. Its reasoning engines next make judgments based on information regarding its database management system. Finally, it delivers the dialogue synthesis control systems graded viable options.  Figure 1 depicts the DSS, which is made up of typical software elements.

Any MCDM choice framework comprises criteria, options, or connections between these facts or rules. Both utility and effectiveness if the choice models are determined by an information gathering procedure. The key resources of information or component pieces of choice models built on the six judgment procedures are introduced in these sections.

## 3. Decision Model

Within the information basic, choice morpho specifies the overall fundamental architecture of a choice paradigm. It consists of two major groups. This collection characteristic is a collection for program assurance traits, while the collection characteristics are a collection of MCDM issue area characteristics.

An excellence product model specifies all program excellence characteristics as well as the connections between the collection of characteristics' components. To describe the collection characteristics, DSS uses both ISO/IEC 25010 standards and the expanded ISO/IEC 9126 standards. These are property computer assurance frameworks that offer benchmark criteria in computer platforms by providing an upper standardized assurance paradigm. Its Product Excellence WOman's components are used to categorize topic characteristics of an MCDM challenge depending on its influence on product solutions options' excellence characteristics.

A domain's descriptions specify the initial and third phases of the judgment procedure, which are represented by the terms “trying to recognize the aim” and “choice of the characteristics”. It describes an MCDM original problem technology makes that translates the collection attributes to the collection characteristics, wherein, Qual*∗*Fea -> Bool, built on the expertise of industry specialists information kind, such as true or numerical, is assigned to every topic characteristic. Domain's characteristics such as fame and a DBMS's firewalls, for instance, be regarded as numerical and binary information kinds, accordingly.

A final phase of the judicial procedure is defined by the showcase, which is represented by the choice of options. This generates a collection of options and translates these to given domains selected characteristics, Alt*∗*Fea -> Bool. Recording on options, literary research, interpersonal connections, rival specialists, and other sources for information might become a primary resource of information during this period.

A fifth phase of the judgment procedure, represented by the weighting technique choice, is defined by the situation formulation. The DSS uses MoSCoW to identify judgment area component needs and rank the significance of those needs. Strong restrictions are industry product needs with softer restrictions are business component needs having priority. In other terms, a scenario description depending on judgment choices (MoSCoW) is a technique to rank area component needs. With numerical realm characteristic needs, the judgment provides desired numbers. In that instance, a judge might consider DBMSs having TCOs of less than USD 5000 to be higher significant over competitors. As a result, a TCO of less than USD 5000 may be deemed a must-have site attribute.

Its information bank is made up of choice types, which are collections of laws and data. The fifth and sixth phases of the judgment procedure, denoted by using the technique for aggregate and judgment depending on aggregate findings, are defined by the reasoning engines. Every domain element needs with priority should be supported by realistic solutions, but necessarily all domain component needs will not want priority. The viable options are ranked by the reasoning machine depending on their computed ratings. Another well-balanced aggregate method is used to calculate the rating. The ultimate ranking of viable answers would be presented as the outcome of the DSS by ranking the plausible alternatives in decreasing sequence of respective ratings.

SPOs have a critical issue in selecting accurate or expensive storage systems. The system architecture (relationship, network, etc.), needed capabilities (operation, backups, etc.), and price are all variables to consider (license, support, etc.). To pick a DBMS that most meets client needs, judgment must use a reliable or repeatable approach. Since this result, SPOs are confronted by an MCDM challenge in determining their appropriate DBMS(s), as they must take a huge amount of comparable judgments. Furthermore, the overall quantity of possible options and selection variables is enormous.

Deciding conceptual, product assurance paradigm, subject descriptions, and showcase are all components of a choice paradigm. The choice concept, which includes two groups comprising quality or characteristics, establishes this fundamental architecture of a choice modeling in the information basic. To describe a collection of characteristics, a judgment paradigm uses criteria ISO/IEC 25010 standards and the expanded ISO/IEC 9126 standards. With choice modeling built upon a DSS methodology, both choice morpho and product model are irreversible. Nevertheless, to create a choice matrix for an MCDM issue, the subject descriptions and showcase must be defined.

To solve the DBMS software purchase difficulty, each part does provide a judgment framework based upon the DSB method. Furthermore, investigations were done to assess the DSS's speed and efficacy in resolving a DBMS decision challenge for SPOs. Subject specialists are the best resource of information for determining the correct collection of subject characteristics, however, documents and literary research on technological alternatives can help to narrow down a preliminary list of subject characteristics. Over 250 parameters were used that describe the overall scope for database management systems choosing challenge (like audits and backups) have been gathered based on industry specialists' recommendations. A product safety framework is a simplified representation of a product integrity paradigm. The topic characteristics are decomposed by this selection engine from abstraction notions. Domain's characteristics must be clearly defined to explain the fundamental value ideas that reflect or relate those to all relevant grade attributes in the collection attributes. Domain characteristics that are not required must be included within a particular grade component by the domain descriptions; domain elements may be found in a variety of grade factors. Instant stability as a DBMS characteristic could perhaps be linked to numerous qualitative elements like raw content and human mistake prevention.

During this research, nine industry specialists in the western Netherlands, comprising three college academics, five computer engineers, and two computer designers, identified industry characteristics and their mappings among both collections attributes with capabilities for a DBMS choice issue. Six tractor-trailer conversations were used to identify area characteristics, and three specialists collaborated in the study to translate the examined topic aspects to a collection of attributes using logical equivalence matrices Qual*∗*Fea -> Bool.

It is necessary to create a table of technological options in the area of concern. A key resource of information for defining the listing of technological options is very well innovation of other products, blogs, relevant forums, or subject specialists. The research looked at 73 DBMS systems spanning 10 different data storage types (relational, document, and so on). Following that, all DBMS technology was tested to see whether they could provide conditional business characteristics. This link is among this category of capabilities and options as specified by both manuals or webpages of the DBMS systems under consideration. Another of its most significant difficulties is the absence of consistent vocabulary in DBMS literature. An identical idea may be referred to by multiple titles by various suppliers, or, much better, the identical term may relate to various notions in distinct DBMS systems.

To avoid conceptual incompatibilities during a DBMS choice phase, it is critical to find discrepancies inside the showcase. Companies frequently show only a portion of their goods. Manufacturers exaggerate marketing advantages on your item while ignoring its flaws, or marketers simply give half the picture. Many quasi-publications evaluate DBMS systems and capabilities, although they were frequently dependent on individual assessors' poor understanding of underlying technology and preferences [11]. Creating evaluation modeling per every quasi-domain characteristic, including marketplace attractiveness and overall price or maintenance, was the following stage in constructing a judgment strategy for your DBMS choice issue.

The findings from these repository rankings are implemented in this research to offer a measure of the industry's attractiveness of DBMS technology. The overall reputation of computer systems is measured by repository employing a variety of key criteria, including the number of references of the systems on web pages and overall curiosity about its platform. This DBMS choice issue's prevalence within the marketplace is a numerical domain characteristic that discovers its more common solutions within its marketplace depending upon judgment' domains characteristic criteria.

The price of DBMS technology varies greatly, ranging from completely cheap to exorbitantly costly, and numerous considerations and alternatives must be evaluated. Databases licenses may be perplexing at moments, particularly while dealing with good suppliers like Java and Windows. In contrast, a wide range of payment techniques or approaches, including each component and each server, was accessible for assessing database subscription prices.

Overall TCO of every option is obtained either through its manufacturer or estimated using TCO calculations available on DBMS supplier webpages. Although most choices, incentives, and inserts are manufacturer exclusive, these are never considered in our TCO estimates. Defining TCO as domains characteristic of defining DBMS choice issue tries to dispel much of that ambiguity surrounding databases licenses. TCO estimates, on business another hand, could potentially offer a complete or exact picture of all complicated payment and license systems used by DBMS suppliers.

During this research, three example investigations were done in the business framework of two SPOs to assess or indicate the overall utility or effectiveness of using DSS for addressing MCDM challenges, especially a DBMS choice issue. While engaging in such studies, the real instance businesses examined a variety of possible DBMS technology within the respective organization via several corporate examinations plus thorough inquiry on DBMS alternatives.

## 4. AFAS Software

AFAS Systems is a Dutch ERP company having over 350 workers. Another problem of AFAS is determining if the company selected the appropriate database management system (DBMS) for the next edition of its primary business. This novel solution combines AFAS QS and AFAS SS for main information retention.

Procure Comp is a computer development company that specialized in purchasing. Windows software is used in Procure Comp's offering. Our Procure Comp software is now getting refreshed and reconstructed on newer Windows systems, and now is a good moment to reconsider the information retention approach for your future Procure Comp edition (NX1). Tables 2 and 3 show the amount of the domains characteristic needs for systems AFAS QS, AFAS SS, or NX1 depending on MoSCoW as reported from instance research respondents.

## 5. Results and Observations

Table 3 shows the DSS's viable answers for AFAS QS, AFAS SS, and NX1. Since Procure Comp specialists limited their searching area via designating 50 domains' characteristics as Should Musts, or tight restrictions, DSS only found 1 viable option for NX1. One cause for this is because NX1's computer design is highly reliant on the relationship between information store and Windows technologies. As a result, Procure Comp's specialists were mainly concerned with determining which version of SQL Servers better met client needs or goals. Its computer design of AFAS is independent of any particular information retention type or provider. Furthermore, many of the major domain functionality needs of AFAS QS and AFAS SS may not need the use of such a unique database management system.

With AFAS, the quantity of yearly TCO was a domain characteristic that must be included. As a result, DSS could be unable to rule out any options depending on respective TCO estimates. Java and IBM DB2 storage technology are never recommended for AFAS, as seen in Table 3. Since the scenario partners discover that the yearly TCO of various DBMS systems, considering additional choices, is significantly greater than the cost of alternative viable alternatives. In other terms, they believe MySQL, SQL Server, and Postgres DBMS technology were appealing alternatives owing to its cheap total cost of ownership (TCO) with such transitional server's setup with more features. Furthermore, according to AFAS specialists, IBM DB2 is unsuitable since people lack the expertise to its achievement, assistance, and licensing.

Its DSS offers excellent options to assist SPOs in making early selections in adopting DBMS technology, according to the example research respondents all at businesses. In other terms, following considerable study or debate, DSS proposed identical remedies to all scenario players' firms. Nevertheless, because the DSS only provides a limited shortlist of all viable options, SPOs need to conduct additional research, including business efficiency tests or real TCO calculations, to choose the best DBMS architecture in particular computer applications. Respondents in this example research claim how respective firms are always improving or reevaluating their technology, particularly any DBMS systems they employ.

Respondents throughout the example research submitted a restricted range of domain characteristic criteria. I was startled to discover out these specialists had a poor understanding of their humankind's industry functionality needs. Moreover, most example partners were startled to learn whatever our major worries appear well become, particularly as the perspectives of many specialists were merged. DSS had sparked debates that have led to technological judgment, demonstrating how it was a viable instrument for both SPOs and TCOS judgment. Moreover, DSS proactive can satisfy even the most esoteric criteria. Most significantly, example research respondents agree how this revised or verified edition of tool DSS is helpful or effective in generating a selection of viable options. Lastly, it cuts down on the amount of both effort and money spent on judgment.

DSS comprises all key elements for a typical DSS, according to all specialists which were contacted. Some also claim that DSS is a valuable instrument because gives them additional information that some would have gathered on their own. Analysts think hands-on contact with a product is essential whenever it comes to picking the right technologies. As a result, they propose suggested DSS be used in conjunction with other standards wherever possible.

At various stages, of a systems engineering existence, SPOs have varied viewpoints on respective subject product needs. Judgment usually evaluates broad topic characteristics initially in its lifespan cycles, but when the software creation cycle continues, people become increasingly engaged in increasingly precise and particular topic characteristics. During a concept stage, for example, entry management may be emphasized as a must has category element, while in that execution step, another of its micro, such as labels driven entry management, may be chosen rather. Moreover, the priority of domain requirements can be modified at any time during the development process. As a result, DSS may provide a variety of remedies for an SPO at several stages of its program production existence. Our suggested DSS is a device that may be employed throughout the entire course or may adapt the recommendations in response to changing needs. Rerunning the judgment procedure is never very consuming since the individuals' selections are saved in a DSS. At the right moment, we're working on methods that will allow “our public” to contribute information while preventing corporate parties from influencing this same information foundation to their benefit. We're also exploring employing textual extraction technologies to dynamically pull topic characteristics like guides or literature.

Judgment may be swayed in the assessments of area features needs and priority. Prejudices, such as emotional and cognition [27], develop as a result of judgment' adoption of conveniences or techniques to resolve issues and complete activities. Another type such mental prejudice includes the Hawthorn impact, which is the propensity of judgment to modify their conduct if it is watched. Since people actively watch other researchers assessing particular chosen area characteristic needs or objectives, scenario research respondents may have acted extra cautious within this laboratory environment than we could be in the actual world. Furthermore, a similar type of mental prejudice was a Rabid fandom, which is a propensity that acts and thinks something when numerous others judgment does or think a similar item. The following impact is most commonly seen in collective choices. Single and team surveys were done to obtain specific area features needed for every specific example to reduce both Harrow or Hype train impacts.

## 6. Conclusion

Whenever it comes to sourcing acceptable TCOs, SPOs confront an MCDM challenge. There are a lot of possible answers (offerings) and decision-making variables. That article is our initial effort for assisting designers with reaching complicated choices, and it is our initial foray into the realm of database management systems (DBMS).

A portal is needed to maintain the DSS's information collection current or accurate come up to build a network surrounding this system which would keep the edited information source out to current on newer DBMS technology and capabilities frequently. That's possible if any DSS application serves as a debate forum, highlighting issues and objectives to draw attention to them and guide the decision-making processes.

In the future scope, this research work can design the groundwork for further research on technological choice challenges. And also this work can be improved with the trusted methodology to handle architecture patterns, potential services providers, and cryptocurrency substrate choosing.

## Figures and Tables

**Table 1:** Compared several MCDM approaches from the literature.

References	Domain	MCDM	Pairwise comparison [yes/no]
[19]	DWS	AHP	N
[21]	ERP	FMCDM	Y
[18]	RMA	FAHP	N
[6]	PDP	FMCDM	N
[11]	COTS	DSS	Y
[20]	DBMS	FTOPSIS	N
[15]	DBMS	FAHP	N

**Table 2:** The number of domain feature needs MoSCoW priority.

MoSCoW	AFAS QS	AFAS SS	NX 1
It is necessary to have	8	5	49
Ought to have	9	5	6
Could've been	8	3	18

**Table 3:** Domain feature needs MoSCoW priorities through the DSS's for AFAS software and ProcureComp.

Study of a case	Feasible solutions	Desirable suggestions	Unfavorable recommendations	DSS score in percent	Rank in CP
AFAS QS	Software packages (my SQL, DB2, oracle, server)	Yes	Yes	100	3
AFAS SS	Software packages (my SQL, DB2, oracle, server)	Yes	No	97	4
NX 1	SQL server	Yes	No	98	2

## Data Availability

The datasets used and/or analyzed during the current study are available from the corresponding author upon reasonable request.
